# Analysis of distribution of DNA methylation in kidney-renal-clear-cell-carcinoma specific genes using entropy

**DOI:** 10.1016/j.gdata.2016.10.008

**Published:** 2016-10-18

**Authors:** Nithya Ramakrishnan, R. Bose

**Affiliations:** Department of Electrical Engineering, Indian Institute of Technology Delhi, Hauz Khas, New Delhi 110015, India

**Keywords:** DNA methylation, Entropy, Cancer, Tumor suppressor genes, Oncogenes

## Abstract

DNA Methylation is an epigenetic phenomenon in which methyl groups are added to the cytosines, thereby altering the physio-chemical properties of the DNA region and influencing gene expression. Aberrant DNA methylation in a set of genes or across the genome results in many epigenetic diseases including cancer. In this paper, we use entropy to analyze the extent and distribution of DNA methylation in Tumor Suppressor Genes (TSG's) and Oncogenes related to a specific type of cancer (viz.) KIRC (Kidney-renal-clear-cell-carcinoma). We apply various mathematical transformations to enhance the different regions in DNA methylation distribution and compare the resultant entropies for healthy and tumor samples. We also obtain the sensitivity and specificity of classification for the different mathematical transformations. Our findings show that it is not just the measure of methylation, but the distribution of the methylation levels in the genes that are significant in cancer.

## 1 Introduction

Epigenetics is the study of heritable physio-chemical changes in the DNA that influence gene expression without changes to the genetic sequence [1]. DNA methylation, histone modifications and microRNA's are some of the significant epigenetic mechanisms. Epigenetic phenomena are known to play a significant role in several metabolic processes of the organism. These biological changes are influenced by external physical factors like environment, stress, diet and light [2].

DNA methylation is an epigenetic mechanism that involves the covalent addition of a methyl group at the 5-carbon of the cytosine ring to result in 5-methyl cytosine (5-mC). In human somatic cells, 5mC occurs in CpG sites and islands. A CpG site is a location within a DNA sequence in which a cytosine and guanine appear consecutively. A CpG island is a long stretch of CpG sites in DNA. When a CpG island in the promoter region of a gene is methylated, the gene expression is turned off. It is also established that DNA methylation affects some physical properties of the DNA like curvature, rigidity and flexibility which may in-turn be related to its transcription inhibition [3].

Abnormal DNA methylation (hypo and hyper methylation) has been associated with many human diseases. In this paper, we focus on cancer, which is considered to be caused by multiple epigenetic events, biomechanical transformations and molecular pattern alterations. Of particular significance are DNA methylation aberrations in the promoter regions of the tumor suppressor genes and oncogenes associated with the specific tumor type [4], [5]. Tumor suppressor genes are normally active in the genome, however the epigenetic silencing of these genes by hypermethylation of DNA in the promoter regions causes these genes to be silenced. Oncogenes, that are silent in the non-cancerous genomes, are found to be “turned on” in cancer, primarily due to hypomethylation of the DNA in the promoter regions [5], [6]. In this paper, we use entropy to analyze the DNA methylation abnormalities in the tumor suppressor genes and oncogenes associated with a specific type of cancer – Kidney Renal Clear Cell Carcinoma (KIRC).

The significance of entropy in the thermodynamic sense in evolution and stability of cells has been established in contemporary research in the field of Constructal law of Physics [7]. Cancer can be regarded as a special case of thermodynamic state transitions [8] with DNA methylation being one of the parameters controlling it. Since Information theoretic entropy is known to model its thermodynamic equivalent in a “subjective statistical mechanics” approach as proposed by Jaynes [9], we seek to analyze the Information theoretic entropy in DNA methylation of specific genes that are biologically significant in cancer.

Several biological and computational techniques have been employed in the past to analyze the associated factors and types of cancer using entropy. In [10], the author uses entropy from statistical thermodynamics to characterize the normal and cancer states for AML (Acute Myeloid Leukemia). He uses maximum entropy distribution on a weighted set of cancer markers to predict AML based on the observed macroscopic properties of its cell populations. In [11], the authors use structural entropy minimization techniques to predict the cancer types based on their gene-maps. For constructing the gene maps, the authors use bio-physical factors such as survival times and other survival scores. Entropy based techniques were used to select the critical genes associated with different cancer types in [12]. The authors use entropy to maximize the relevance and minimize the redundancy in the selection of the genes. In [13], the authors study splice variants specific to cancer genes using entropy. They show that splice disorders are particularly common in cancer tissues using entropy ratios.

There have also been several papers exploring DNA methylation and its effect on cancer using physical and mathematical approaches. The authors introduce a quantitative measure for methylation in differentially methylated regions (DMR's) in [14]. In the same paper, the authors define entropy based on methylation level in a region of a sample relative to the total value in all samples for that region. However the authors do not study the methylation levels of specific genes like Tumor Suppressor Genes or Oncogenes. In a related research [15], the authors define ‘Methylation Entropy’ based on methylation patterns in contiguous CpG nucleotides and make genome wide assessments for both normal and cancer cells. The authors do not focus on methylation level intensities or in the prediction of cancer based on specific genes but make genome wide observations.

There has also been research on the biological and mathematical analyses of a specific kind of cancer. In [16], the authors study the genes and the pathways associated with Kidney Renal Clear Cell Carcinoma (KIRC). They use Support Vector Machines (SVM's) to predict the state of unknown samples and ROC curves to rate the effectiveness of classification. It has to be noted that the authors use the TCGA database (The Cancer Genomic Atlas) database [17] to extract the KIRC data and provide a comprehensive list of genes (including TSG's and oncogenes) associated with KIRC. However they do not specifically explore the entropy of DNA methylation of TSG's or oncogenes in their work. In [18], the authors discuss various classification models and their performances as applied to the KIRC RNA data obtained from the TCGA database. However, they do not focus on the DNA methylation data or TSG's and oncogenes in KIRC.

In this paper, we propose to provide a mathematical and bio-physical perspective of how DNA methylation in specific sets of genes (Tumor Suppressor Genes and Oncogenes) can help in the prediction of cancer in accordance with the literature in cancer epigenesis [5], [6]. We define entropy in the context of the probabilistic randomness of DNA methylation for a set of genes and use the measure to compare the significance of the intensities of methylation in cancer prediction. Since DNA Methylation changes are linked with the physical properties of the DNA, the entropy measure would estimate the bio-physical implications in the cancer analysis. We also use mathematical transformations on the methylation level probabilities to enhance different ranges and compare the prediction sensitivities. We show that the distribution of methylation levels in the set of genes is more significant than just the intensity of methylation levels in the occurrence of cancer. In this paper, we focus on KIRC, a fatal cancer type of the renal and associated tissues [16].

## 2 Methods

### 2.1 Specific entropy for DNA methylation

As in the case of [10], we begin with the definition of Shannon entropy:

(1) $$H\left(X\right)=-\sum_{i=1}^{N}p\left(x_{i}\right)\ln p\left(x_{i}\right)$$

In Eq. (1), *X* is a discrete random variable with possible values in the alphabet {*x*_*1*_, *x*_*2*_, …, *x*_*N*_} and *p*(*x*_*i*_) represents the probability of *x*_*i*_. When the base of the logarithm is 2, *H*(*X*) is measured in bits.

We now consider the methylation levels as obtained from the Level 3 Illumina27K chip of the TCGA database. For this data, we define the alphabet for methylation entropy computation {*C*_*i*_} as follows:

(2) $$\zeta =\left\{C_{1},C_{2},C_{3},C_{4},C_{5},C_{6},C_{7},C_{8},C_{9},C_{10}\right\}$$

In Eq. (2), *C*_1_–*C*_10_ represent the symbols corresponding to the discretized methylation intensities of a CpG site as elaborated in Table 1. It has to be noted that the no. of bins for discretization was chosen as 10 (corresponding to the 10 symbols in the alphabet in Eq. (2)) as an optimum measure with values for the data under consideration, but this can be regarded as a design parameter subject to change based on the a different dataset (chip or the different levels of methylation). The methylation levels are defined as the intensities of the probes in the Illumina27K chips. Bio-physically, the levels can be interpreted as a measure of methylation (either in both or single strands of DNA) for the specific genes across the genome. In our experiments, we consider only those CpG sites which correspond to the known TSG's or Oncogenes for that specific kind of cancer. To analyze global methylation changes, this pre-processing step can be skipped and all the CpG sites available for the sample can be considered.

As the next step, the methylation probabilities (***P***) in a given methylated sample are computed as

(3) $$p_{i}=N_{i}/N$$

where *p*_*i*_ represents the probability of occurrence of symbol *C*_*i*_ enlisted in Eq. (2), *N*_*i*_ is the frequency of occurrence of the symbol *C*_*i*_ in the sample. *N* is the number of CpG sites considered in the sample. We use Eq. (1) on the probabilities (*p*_*i*_) to compute the specific entropy of methylation (*H*_*m*_) for the given set of genes in a sample. This quantity represents the measure of randomness of DNA methylation levels across a specific set of genes for a sample. This definition of entropy differs from the previously defined quantity in literature [14], [15] in that it focuses on methylation levels and can be applied on specific set of genes to analyze their impact on cancer.

### 2.2 Mathematical transformations

**Fig. 1:**
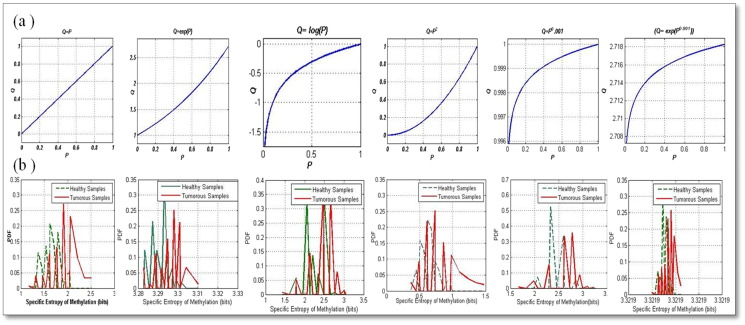
Plots of the (a) transformations and the (b) corresponding modified methylation entropies corresponding to the tumor suppressor genes for the healthy and tumor samples obtained from the TCGA database. We can observe that for the gamma transformation (***Q*** = ***P***^0.001^) that enhances the lower order probabilities; the overlap of the healthy and tumor PDF curves is less.

In order to analyze the significance of distribution of methylation levels, we propose a novel approach where the probabilities of the methylation levels are transformed using suitable mathematical functions to enhance or suppress certain regions of methylation levels. The resultant values are normalized to yield the modified methylation probabilities (***Q***). It has to be noted that this technique applies to individual samples and is not dependent on the a priori knowledge about the nature of the sample. This transformation process is represented mathematically in Eq. (4). Using (***Q***), the modified specific methylation entropies are calculated and compared for different mathematical functions. The first row in Fig. 1 shows how the transformations help to enhance the different ranges of probabilities. As an example, the logarithm transformation enhances the lower order probabilities while the exponential enhances the higher order probabilities.

(4) $$Q=T\left(P\right)$$

We then train a classifier (Naïve Bayes) to predict samples from the test data based on their entropies for different transformations. We calculate the true positives (*tp*), false negatives (*fn*), true negatives (*tn*) and false positives (*fp*) after the classification process. The definitions for these parameters are provided in Table 2. The performance of the classifier is computed using the sensitivity and specificity defined using Eqs. (5), (6). Sensitivity can be understood as a measure of how accurately the proposed method of classification can identify a valid case of tumor while specificity is a measure of how reliably the method can ignore the case of false positives.

(5) $$\mathrm{Sensitivity}=\frac{\mathrm{tp}}{\left(\mathrm{tp}+\mathrm{fn}\right)}$$

(6) $$\mathrm{Specificity}=\frac{\mathrm{tn}}{\left(\mathrm{tn}+\mathrm{fp}\right)}$$

For processing the data and running the algorithms, Matlab program functions were used. The data was converted to the required format (MS Excel) and the necessary values were read using the programs. The histogram computations were also based on Matlab software. The Naïve Bayes classifier was chosen with the standard Gaussian filter (the default parameter to the Matlab NaiveBayes routine). The data for test and training samples were chosen randomly and the results were averaged over 5 trials.

## 3 Results

### 3.1 Data Extraction and processing

We extracted the relevant data from the TCGA database [17] with the following filter settings in the Data matrix: Disease: KIRC (Kidney renal clear cell carcinoma), Data type – DNA Methylation, Data Level – Level 3, Tumor/Normal checkbox – Tumor Matched or Normal Matched for Tumor/Healthy Samples, the other parameters were the default settings. Only the Illumina27K DNA Methylation samples were taken for our experiments. We obtained about 200 healthy and 219 tumor samples. Each sample consisted of CpG sites with their corresponding gene symbols and beta values (methylation intensities). We used Matlab software in processing the samples. Matlab routines were coded to convert the data files into the appropriate format for processing. The sequences with beta values listed as ‘NA’ were ignored in our computations.

Only those CpG sites that corresponded to the Tumor Suppressor Genes or Oncogenes identified for KIRC were considered for further analysis. This shortlisting of the required CpG sites was also achieved using Matlab software programs. As mentioned in the Methods section, this step can be skipped if the global analysis is to be performed. The lists of Tumor Suppressor Genes and Oncogenes for KIRC that were obtained from [16] are provided in the Supplementary information.

### 3.2 Tumor suppressor genes

First, we consider the results and observations for the data processed for the tumor suppressor genes. The data was split into training data (70%) and test data (30%) randomly and all the results were averaged across 5 trials. The mean of methylation intensities was computed for all the healthy and tumor samples. The mean value across the healthy samples was 0.2706 and across tumor samples, it was computed to be 0.2670. From these values, we understand that the healthy and tumor methylation intensities are not widely separated and statistical means might not be efficient in separating them. To corroborate this inference, we trained a Naïve Bayes classifier for the data based on statistical means. We obtained a sensitivity of 0.5976 and specificity of 0.7500. These cannot be considered to be very high.

The Matlab functions *NaiveBayes.fit* and *predict* were used for training and prediction of the classifier. The default Gaussian distribution was used in parameterizing the NaiveBayes functions. These functions and parameters of the classifier were employed in the case of the oncogenes and the global data set as well.

Next we computed the entropies of the methylation intensities of the training healthy and tumor samples using Eq. (1) without any mathematical transformations on the probabilities (***Q*** = ***P***). The mean of the entropies of the healthy samples is computed to be 1.6569 bits while the mean of the entropies of the tumor samples is computed to be 1.9054 bits. The higher values of specific entropy of methylation (in TSG's) of tumor samples indicates that there is a higher degree of randomness in the methylation intensity distribution across the tumor suppressor genes in case of tumor than in healthy samples. The Naïve Bayes classifier (with Gaussian distribution) trained based on this data yielded a sensitivity of 0.7231 and a specificity of 0.8113 which are much higher than those obtained with the statistical means listed above. These values can be observed from the first row of Table 3 which corresponds to the no transform case (***Q*** = ***P***).

To analyze the methylation intensity distributions further, we apply mathematical transformations on the probabilities of the methylation intensities for the tumor suppressor genes. Table 3 lists the sensitivity and specificity values of the classifier for the different transformations. We observe that when the lower order and higher order methylation intensities are enhanced as in the case of log, exponential and *p*^0.01^ transformations, the classification measures are much higher. The highest sensitivity is obtained for the (***Q*** = ***P***^0.01^) transformation (0.8740) followed by the log transformation (0.7708). The highest specificity was obtained for the exponential transformation (0.8824). These values can be inferred from Table 3.

### 3.3 Oncogenes

Next, we consider the results for the oncogenes corresponding to KIRC tumor. Similar to the tumor suppressor genes approach, the data was split into 70% training and 30% test data. A Naïve Bayes classifier trained with the means of the methylation intensities of the healthy and tumor samples yielded a sensitivity of 0.6373 and a specificity of 0.5392, which are not very high indicating that the methylation distributions for oncogenes cannot be statistically segregated for the healthy and tumor samples.

The specific entropy of methylation for oncogenes for the healthy training data was computed as 0.3159 bits while the corresponding to the tumor training data was computed as 1.3265 bits. On applying the transformations to the methylation probabilities, we observed that most of the methylation intensities were spread in the lower order probabilities and when these were enhanced, better sensitivity results were obtained. The highest sensitivity results were obtained (0.9837) for the ***Q*** = log(***P***^0.001^) transformation. These can be observed in Table 4. These high values of sensitivity and specificity provide clues to how specific transformations of probabilities of methylation distribution help to segregate the healthy and tumor samples more efficiently.

## 4 Discussion

From these results, one can observe that there are the key methylation intensities distributed in the specific ranges and when these are enhanced with suitable transformations like logarithm, exponential or gamma, the minor differences in the patterns of the entropy variations between the healthy and tumor samples are highlighted, leading to better classification. Bio-physically, this can be interpreted as a higher degree of randomness in the lower order methylation levels of the cancer-significant genes. This also leads to an inference that it is not just the measure of methylation in the genes but the distribution of methylation that is important in cancer.

It has to be noted that this approach and the resultant comparison values are based on DNA methylation data from the TCGA database as opposed to the RNA sequencing data in the previous approaches [16], [18]. The computational complexity is also quite less in this approach – the average time taken to compute the entropy using the above technique for a given sample was 0.380 s while the average time for pre-processing (narrowing down the CpG sites corresponding to specific genes) is about 10.025 s for a single global methylation sample file. As noted previously if the global DNA methylation entropies are to be analyzed, the pre-processing step can be skipped.

**Fig. 2:**
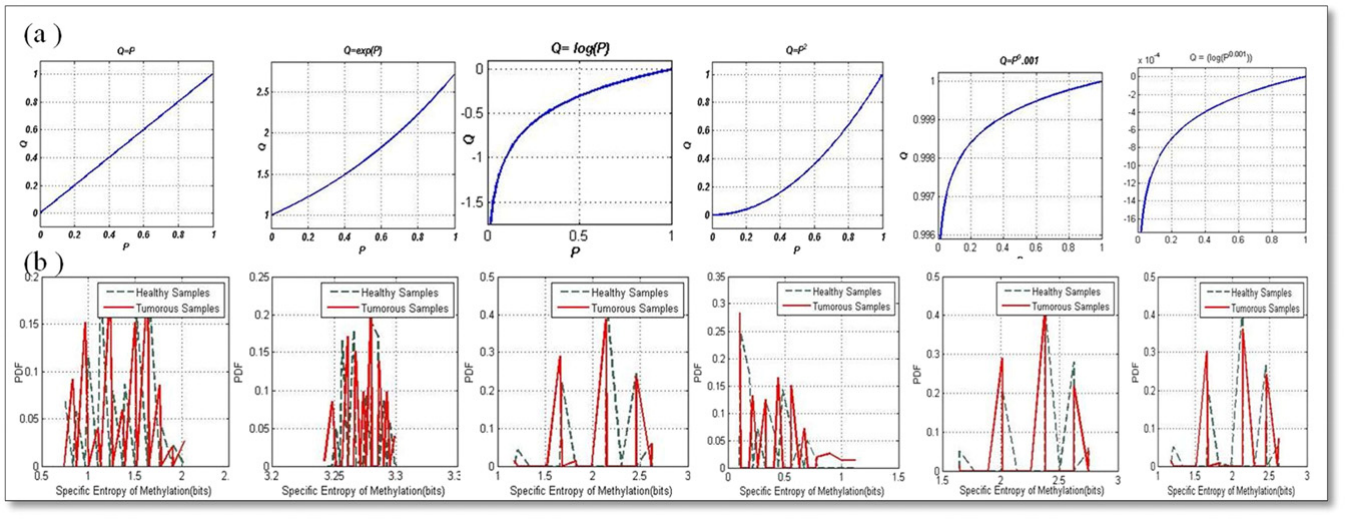
Plots of the (a) transformations and the (b) corresponding modified methylation entropies corresponding to oncogenes for the healthy and tumor samples obtained from the TCGA database. We can observe that for the log and gamma transformations that enhance the lower order probabilities, the overlap of the healthy and tumor PDF curves is less.

Fig. 1 shows the (a) various transformations applied to the methylation probabilities and the (b) corresponding PDF's of the resultant modified specific entropies of methylation for the tumor suppressor genes for both the healthy and tumor samples. We can observe from the figure that for the *P*^0.01^ transformation, the PDF curves are less overlapped which can be correlated with the higher sensitivity values for this transformation from Table 3. Fig. 2 shows the (a) various transformations applied to the methylation probabilities and the (b) corresponding PDF's of the resultant modified specific entropies of methylation for the oncogenes for both the healthy and tumor samples. We can observe from the figure that for the log(*p*^0.001^) transformation, the PDF curves are least overlapped which can be correlated with the higher sensitivity values for this transformation from Table 4.

## 5 Conclusion

To conclude, we have analyzed the entropy of DNA methylation data of specific sets of genes that are significant in cancer. We have proposed techniques based on this entropy to classify healthy and tumor samples based on the DNA Methylation data for KIRC cancer with samples obtained from the TCGA database for specific TSG's and Oncogenes. We have applied different transformations to the methylation probabilities to study different ranges of entropies with the corresponding PDF's and obtained the classification results. It has to be noted that the range of mathematical transformations are not limited to the ones tested in our case and can be varied for different sets of genes for other cancer types to enhance significant regions of entropy. Nonetheless, with our observations on the given dataset and the chosen genes, we infer that the distinctive regions of methylation lie in the lower order of methylation intensities leading to significant entropy differences for healthy and tumor samples. We believe that this can be used as a valuable tool in the early prediction of cancer using the DNA methylation data and the cancer-significant genes associated with the specific cancer type.

## Conflict of interest

The authors declare no conflict of interest with any person or organization as part of this research.

## Figures and Tables

**Table 1:** Mapping of methylation intensity levels and the corresponding symbols for the Level 3 Illumina27K TCGA data.

Methylation levels – beta value in the samples	Symbol
0 < *i* ≤ 10	*C*_1_
10 < *i* ≤ 20	*C*_2_
20 < *i* ≤ 30	*C*_3_
30 < *i* ≤ 40	*C*_4_
40 < *i* ≤ 50	*C*_5_
50 < *i* ≤ 60	*C*_6_
60 < *i* ≤ 70	*C*_7_
70 < *i* ≤ 80	*C*_8_
80 < *i* ≤ 90	*C*_9_
90 < *i* ≤ 100	*C*_10_

**Table 2:** Definition of parameters in the calculation of sensitivity and specificity.

	Decoded as healthy	Decoded as tumor
Healthy phenotype	True negative (*tn*)	False positive (*fp*)
Tumor phenotype	False negative(*fn*)	True positive (*tp*)

**Table 3:** Tabulated results of sensitivity and specificity of classification for the KIRC DNA methylation data of Tumor Suppressor Genes for healthy and tumor samples obtained from TCGA database.

Transform	Sensitivity	Specificity
***Q*** = ***P***	0.7231	0.8113
***Q*** = log(***P***)	0.7708	0.7308
***Q*** = exp(***P***)	0.7105	**0.8824**
***Q*** = ***P***^2^	0.6104	0.7400
***Q*** = ***P***^0.01^	**0.8740**	0.7024
***Q*** = exp(***P***^0.001^)	0.7391	0.8305

**Table 4:** Tabulated results of sensitivity and specificity of classification for the KIRC DNA methylation data of Oncogenes for healthy and tumor samples obtained from TCGA database.

Transform	Sensitivity	Specificity
***Q*** = ***P***	0.4219	0.4835
***Q*** = log(***P***)	0.7833	0.5345
***Q*** = log(***P***^0.001^)	**0.9837**	**0.8537**
***Q*** = exp(***P***)	0.5143	0.5376
***Q*** = ***P***^2^	0.5	0.7682
***Q*** = ***P***^0.001^	0.8833	0.7345

## Appendix A Supplementary data

Supplementary material Image 1
