# Supplementary material for: Analysis of distribution of DNA methylation in kidney-renal-clear-cell-carcinoma specific genes using entropy
Source: Genom Data. 2016 Oct 18;10:109–13. doi: 10.1016/j.gdata.2016.10.008 (PMC5080556; doi:10.1016/j.gdata.2016.10.008)
Supplement: Supplementary file 1 — Supplementary material [file mmc1.docx]

**SUPPLEMENTARY INFORMATION**

**Analysis of distribution of DNA methylation in kidney-renal-clear-cell-carcinoma specific genes using entropy**

Nithya Ramakrishnan* and R.Bose

*Department of Electrical Engineering, Indian Institute of Technology Delhi, Hauz Khas, New Delhi – 110015*

*Correspondence to email address *nithya.ramakrishnan@ee.iitd.ac.in*

Supplementary Data S1: List of Tumor Suppressor Genes for KIRC disease

| KANK1 |
| --- |
| PKD1 |
| RBM5 |
| EPHB2 |
| GTPBP4 |
| PRR5 |
| TP53BP2 |
| TP53 |
| BLM |
| VHL |
| PBRM1 |
| SETD2 |
| BAP1 |
| PTEN |
| TCEB1 |
| DLEC1 |
| RARB |
| LIMD1 |
| MLH1 |
| TUSC4 |
|  |

Supplementary Data S2: List of Oncogenes for KIRC disease

| ERBB4 |
| --- |
| KRAS |
| HRAS |
| MET |
| MYC |
| KIT |
| FUS |
| ECT2 |
| AKT3 |
| MFNG |
| MYB |
| ETV4 |
